# Mechanical investigations of the peltate leaf of *Stephania japonica* (Menispermaceae): Experiments and a continuum mechanical material model

**DOI:** 10.3389/fpls.2022.994320

**Published:** 2023-01-27

**Authors:** Domen Macek, Hagen Holthusen, Annabell Rjosk, Stephan Ritzert, Thea Lautenschläger, Christoph Neinhuis, Jaan-Willem Simon, Stefanie Reese

**Affiliations:** ^1^ Institute of Applied Mechanics, Rheinisch-Westfälische Technische Hochschule (RWTH) Aachen University, Aachen, Germany; ^2^ Institute of Botany, Technische Universität Dresden, Dresden, Germany

**Keywords:** *Stephania japonica*, mechanical investigations, material model, viscoelasticity, anisotropy, finite strains

## Abstract

*Stephania japonica* is a slender climbing plant with peltate, triangular-ovate leaves. Not many research efforts have been devoted to investigate the anatomy and the mechanical properties of this type of leaf shape. In this study, displacement driven tensile tests with three cycles on different displacement levels are performed on petioles, venation and intercostal areas of the *Stephania japonica* leaves. Furthermore, compression tests in longitudinal direction are performed on petioles. The mechanical experiments are combined with light microscopy and X-ray tomography. The experiments show, that these plant organs and tissues behave in the finite strain range in a viscoelastic manner. Based on the results of the light microscopy and X-ray tomography, the plant tissue can be considered as a matrix material reinforced by fibers. Therefore, a continuum mechanical anisotropic viscoelastic material model at finite deformations is proposed to model such behavior. The anisotropy is specified as the so-called transverse isotropy, where the behavior in the plane perpendicular to the fibers is assumed to be isotropic. The model is obtained by postulating a Helmholtz free energy, which is split additively into an elastic and an inelastic part. Both parts of the energy depend on structural tensors to account for the transversely isotropic material behavior. The evolution equations for the internal variables, e.g. inelastic deformations, are chosen in a physically meaningful way that always fulfills the second law of thermodynamics. The proposed model is calibrated against experimental data, and the material parameters are identified. The model can be used for finite element simulations of this type of leaf shape, which is left open for the future work.

## 1 Introduction

The plant leaf is exposed to a variety of mechanical influences including environmental stresses and stresses from self-loading (Niklas, 1992; Niklas, 1999). The leaf must withstand these stresses and twist and bend without being damaged to secure its function as a platform for photosynthesis and production of organic compounds (Niklas, 1999; Adams and Terashima, 2018). Plants have evolved a multitude of different leaf shapes with many of them being composed of petiole and lamina. From a mechanical perspective, the petiole can be defined as a cantilevered beam fixed on one side and the free moving lamina attached to the other (Niklas, 1999). The lamina itself is composed of a scaffold of leaf veins supporting and supplying the intercostal areas in between that consist of mesophyll which is the main photosynthetic tissue of the leaf (Adams and Terashima, 2018). The internal composition and organisation of different tissues define the mechanical properties of the structural elements of the leaf and thus, its stability (Niklas, 1992). The thin-walled parenchyma and the collenchyma with partially thickened cell walls are hydrostatic tissues. Their mechanical behavior is greatly affected by turgor pressure (Falk et al., 1958; Niklas, 1992). In contrast, fibers and xylem elements are sclerenchymatous, non-hydrostatic tissues. They are characterized by thickened, lignified cell walls and elongated shape (Napp-Zinn, 1973). The polymer lignin within the cell walls of these tissue types acts as additional reinforcement resulting in significantly higher elastic moduli than parenchyma and collenchyma (Niklas, 1992; Gibson, 2012).

While anatomical and biomechanical properties of leaves are generally well studied, not many research efforts have been devoted to investigate the peltate leaf shape. Peltate leaves are defined by the petiole inserting on the abaxial side of the lamina resulting in a 3-dimensional spatial arrangement (Troll, 1932; Langer et al., 2021b; Wunnenberg et al., 2021). The peltate leaf shape is not very common in the plant kingdom. The most recent taxon list comprises approximately 350 peltate-leaved species representing 99 genera and 40 families (Wunnenberg et al., 2021). The 3D architecture of this leaf shape determines the stresses the leaf must withstand, especially in the petiole and the transition zone between petiole and lamina. Petioles of peltate leaves need to be resistant or flexible to bending and torsion in the same intensity in all directions due to the 3D spatial arrangement of the lamina. In comparison, leaves with a 2D configuration need to be resistant in bending in one direction while still being flexible in torsion (Langer et al., 2021a). The transition zone is characterized by a significant change in geometry from petiole to lamina, plays a crucial role in the dissipation of mechanical loads and is optimized to cope with different loads in comparison to the petiole (Sacher et al., 2019; Langer et al., 2021b; Langer et al., 2022).

An anatomical study on peltate leaves (Wunnenberg et al., 2021) already showed a complex fiber organisation in the petiole-lamina transition zone of two *Stephania* Lour. species. In *Stephania delavyi* Diels and *Stephania venosa* (Blume) Spreng., a ring-like structure was described for the transition zone making this genus especially interesting for further analysis.

*Stephania japonica* (Thunb.) Miers is a slender climbing plant species with peltate leaves that belongs to the Menispermaceae. It is native to tropical and subtropical Asia and Australia and grows in hedges, thickets, secondary growths, forests and along river banks at altitudes from 0 to 2000 m (Forman, 1986; Dataset POWO, 2022). It has herbaceous or woody stems that can grow to heights of 10 m. The leaves are petiolate. The petiole is 3 to 12 cm long and can be glabrous or pilose. The lamina is triangular-ovate to ovate in shape with acuminate apex and broadly rounded base and 4 to 17 × 4 to 14 cm in size. The adaxial side of the lamina is glabrous while the abaxial side can be glabrous or pilose (Forman, 1986).

Experiments have shown that the uniaxial tensile nonlinear stress-strain response of petiole, venation and intercostal area is rate dependent. Furthermore, anatomical investigations of the leaf have led to the assumption that the plant tissue is a fiber-reinforced material with one fiber direction. To model the mechanical behavior of such plant tissues, a number of complex features including finite deformation, anisotropy and viscoelasticity have to be taken into account. Although multi-scale methods can be helpful in describing such materials (see e.g. Stier et al., 2015), we focus here on the computationally cheaper macro-mechanical approach.

Several phenomenological approaches for fiber-reinforced composites have been proposed. For instance, Holzapfel and Gasser (2001) introduced a viscoelastic model for two groups of fibers. Nedjar (2007) and Nguyen et al. (2007) presented independently two models that treat the matrix and the fibers separately allowing as many bundles of fibers as desired. Latorre and Montáns (2015) presented an extension of the Reese and Govindjee (1998) framework to quasi-incompressible transversely isotropic and orthotropic materials, later they Latorre and Montáns (2016) presented a formulation based on a reverse (to that from Sidoroff) multiplicative decomposition of the deformation gradient. Furthermore, Liu et al. (2019) provided a model, based on the Holzapfel type anisotropic hyperelastic strain-energy function, with a new method to develop the evolution equations of the viscous internal variables.

Some works have explicitly focused on developing models for soft biological tissues. Bischoff et al. (2004) provided a rheological model for fibrous tissue, Nguyen et al. (2008) presented a constitutive model for the corneal stroma. Garcia-Gonzalez et al. (2018) developed a framework for the mechanical modelling of a wide variety of soft tissues that incorporates strain rate and temperature dependencies as well as the transverse isotropy. Moreover, Stumpf (2021) proposed a constitutive framework capable of modeling a wide variety of different soft tissues, such as skin, tendon, colorectal tissue and brain white matter.

However, according to the knowledge of the authors, an experimentally based sophisticated model for the physical mechanisms of the plant leaf as a whole and the peltate leaf of *S. japonica* cannot be found in the literature.

In perspective of possible engineering applications, in this study we aim to give a detailed anatomical and mechanical description of the peltate leaf of *S. japonica*. To simulate the mechanical behavior of the petiole, venation and intercostal areas, we present a constitutive model of transversely isotropic viscoelasticity at finite deformations. The model is based on a general phenomenological approach for fiber-reinforced composites, since leaf components are considered as a continuum mixture consisting of fibers orientated in one direction and embedded in a soft isotropic matrix. The model is based on a decomposition of the deformation gradient into elastic and viscous parts. Furthermore, we postulate the Helmholtz free energy, which is decomposed into isotropic and anisotropic parts. Both parts are additionally split into elastic and inelastic components. Here, we propose that the viscoelastic behaviors of the matrix and fibers mutually contribute to the nonlinear viscoelastic response of the leaf structures.

## 2 Materials and methods

### 2.1 Plant material and sampling

**Figure 1:**
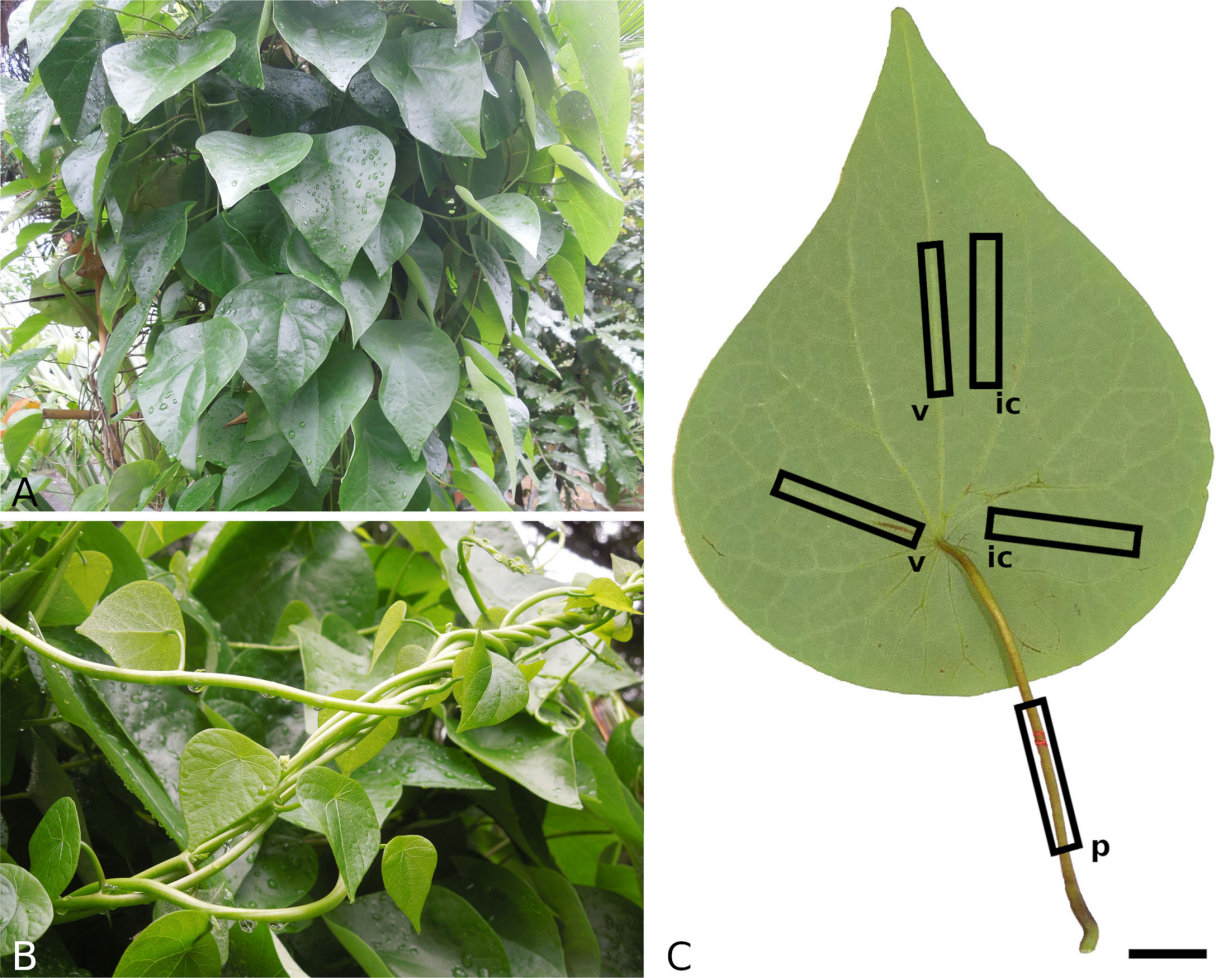
Growth habit of *Stephania japonica* **(A, B)** and sampling method for mechanical testing **(C)**. Petiolate leaves with a triangular-ovate to ovate lamina **(A)** and young climbing shoots **(B)**. Examplary positions and orientations of leaf samples for mechanical tests **(C)**, v - venation, ic - intercostal area, p - petiole, scale: 2 cm.

Leaf samples of *S. japonica* were provided by the Botanical Garden of Technische Universität Dresden, Germany. The plant (**Figures 1A, B**) is cultivated in a pot under open air conditions during summer and in the greenhouse during winter. Only intact, undamaged adult leaves were collected and transported in airtight containers. The fresh leaves were photographed (Lumix DMC-G81, Panasonic, Kodama, Osaka, Japan) from both sides and subsequently processed within a few hours or preserved in ethanol ( 70 *%* ) for later analysis.

### 2.2 Anatomy and morphology

The software ImageJ (National Institutes of Health, Bethesda, Maryland, USA) was used for measurement of leaf and petiole dimensions for all samples. Leaf length was determined at the longest point along the midrib, leaf width perpendicular to the midrib at the widest part. MS Excel (Microsoft Corporation, Redmont, Washington, USA) was used for compilation of measured parameters.

Transverse sections of the petiole and transverse and longitudinal sections of the petiole-lamina transition zone were prepared with a razor blade or vibratome (Hyrax V50, Carl Zeiss AG, Jena, Germany). Sections were bleached in sodium hypochlorite solution (2.8 %), washed in water and stained with astrablue/safranin. Astrablue stains non-lignified tissue blue and safranin stains lignified tissue red. Subsequently, the sections were differentiated in ethanol (70 %). The stained sections were photographed using a light microscope (VHX-970F, Keyence AG, Osaka, Japan) and integrated camera (VHX-970F, CMOS-image sensor, Keyence AG, Osaka, Japan). The amount of lignified tissue in the petiole was measured for three large leaves. For each of these leaves, five sections from basal, central and apical part of the petiole were analyzed using the software ImageJ (National Institutes of Health, Bethesda, Maryland, USA). Following data analysis was performed using OriginLab 2021 Pro (OriginLab Corporation, Northhampton, Massachusetts, USA).

For X-ray tomography, the petiole-lamina transition zone was cut out of a fresh leaf using a razor blade (sample dimensions: 10 mm in height). The lamina was cut away from the transition zone without damaging the latter. Afterwards, the sample was wrapped in several layers of parafilm (Bemis Inc., Neenah, USA) and glued (UHU instant glue, UHU GmbH & Co. KG, Bühl, Germany) to a small wooden sample holder (dimensions: 5×5 mm ). Tomography measurements were done on the micro-CT ProCon CT-XPRESS (ProCon X-Ray GmbH, Sarstedt, Germany) and analyzed using the software Dragonfly 3.5 (Object Research Systems Inc., Montreal, Canada). The axial CT scans were performed with a source voltage of 30 kV and a source current of 70 µA (voxel size: 2.5 µm).

### 2.3 Mechanical tests

Individual samples of petiole, venation and intercostal areas were subjected to displacement driven tensile and compression tests with three cycles on different strain levels. The compression tests were only performed on petiole samples. Samples of 8 leaves in total were prepared and tested (for sample orientation, see **Figure 1C**). For tensile testing, petioles were cut into pieces of 50 mm . The venation was separated from intercostal areas using a razor blade and cut to a length of 50 mm . The intercostal areas were cut into 5×50 mm samples. For compression tests, samples of 2 mm length were cut from the petioles. Width of petiole and venation samples and thickness of intercostal area samples were measured using a digital caliper (Precise PS 7215, Burg-Wächter, Wetter-Volmarstein, Germany). Per testing speed, five samples of each structural element were tested. To minimize water loss, the samples were stored between wet paper sheets until testing and sprayed with water during testing.

**Figure 2:**
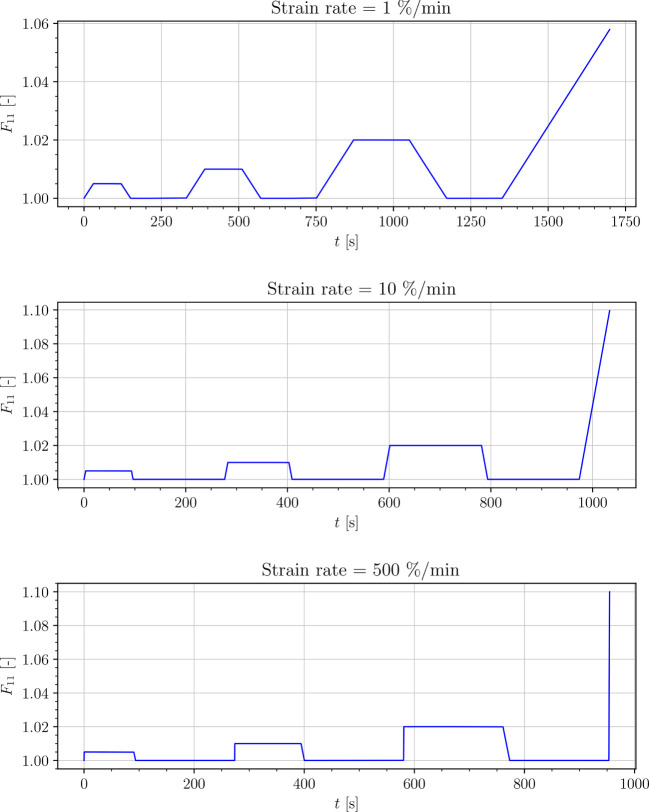
Strain-driven cyclic experiments.

**Figure 3:**
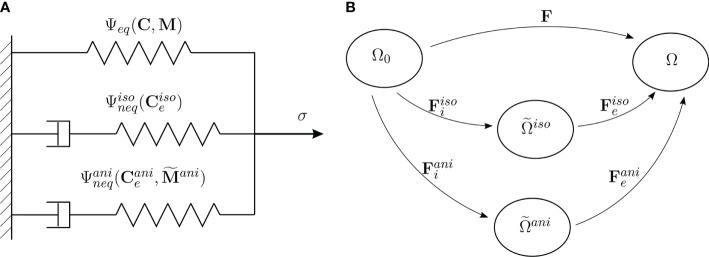
**(A)** Rheological model. **(B)** Initial, current and intermediate configurations of a body, and the multiplicative decomposition of the deformation gradient F.

Two universal testing machines (zwickiLine and Zwick/Roell AllroundLine Z005, Zwick/Roell GmbH & Co. KG, Ulm, Germany) and corresponding software (testXpert II V3.5, Zwick/Roell GmbH & Co. KG, Ulm, Germany) were used. Clamping length was set to 20 mm; pre-load was set to 0.1 N for petiole samples and 0.05 N for venation and intercostal area samples. Resulting forces were recorded using a 50 N load cell for the zwickiLine (Type: KAP-Z, AST Angewandte Systemtechnik GmbH, Dresden, Germany) and a 5 kN load cell for the AllroundLine Z005 (Zwick/Roell xforce P, Zwick/Roell GmbH & Co. KG, Ulm, Germany). Tests were performed at three testing speeds: 1 % min^−1^, 10 % min^−1^ and 500 % min^−1^. The engineering strain (*ε*: = *u*/*l*_0_) levels for the three cycles were set to: 0.5 %, 1 % and 2 %. In each cycle the engineering strain was applied using a ramped constant rate load function. After the prescribed engineering strain value was achieved, the engineering strain was held for the some loading time, as presented in **Figure 2**, to show the relaxation process of the tissue. Next, the engineering strain was removed at a constant rate (see **Figure 3**). A small initial force was applied at the start in order to straighten the specimens. Tests were limited with the total testing time of 30 min, since the samples were drying out during the experiment.

Force-displacement measurements were obtained. For an objective comparison of experimental data, strain and stress measures have to be introduced. Since only uniaxial experiments were performed, stresses and strains can be computed in the following way


(1) $${\left[\text{F}\right]}_{11}:=\frac{u+l_{0}}{l_{0}},$$

(2) $${\left[\text{P}\right]}_{11}:=\frac{R}{A},$$

where *R* is the measured force, *A* is the initial cross sectional area of the specimen, *l*_0_ is the initial length of the sample, and *u* is the measured displacement. **F** is the deformation gradient and **P** is the first Piola-Kirchhoff stress tensor (see Section 2.4 for other definitions).

### 2.4 Material model formulation

In the following, we develop a continuum mechanical transversely isotropic viscoelastic material model at finite deformations to model the behavior of the plant tissues. The three structural elements of the *S. japonica* leaf (petiole, venation, intercostal area) are represented as a continuum mixture consisting of one group of fibers bound by an isotropic matrix. To model the behavior of the tissue, we postulate a Helmholtz free-energy. The stress response of the tissue is schematically described by the rheological model shown in **Figure 3A**. In this rheological model, a single independent spring represents the time-independent equilibrium response which acts in parallel to two Maxwell elements representing two different time-evolving nonequilibrium processes. We assume that the time-dependent behavior of the tissue is dominated by both, the isotropic viscoelastic deformation of the matrix and the anisotropic viscoelastic deformation of the fibers. Thus, the equilibrium response of the tissue, represented in **Figure 3A** by the equilibrium spring, includes isotropic and anisotropic elastic behavior. The nonequilibrium response of the isotropic matrix is represented by the first Maxwell element in **Figure 3A** and the nonequilibrium response of the anisotropic part (fibers) is represented by the second Maxwell element.

#### 2.4.1 Kinematic assumptions

The kinematics of the nonequilibrium process of the whole tissue is described by the multiplicative split of the deformation gradient **F**:=∂ ***χ*** (***X***,*t*)/∂ ***X*** (***χ*** (***X***,*t*) motion, ***X*** position vector w.r.t. undeformed configuration) into elastic and inelastic (viscous) parts (see Reese and Govindjee, 1998; Nguyen et al., 2007),


(3) $$\text{F}={\text{F}}_{e}^{iso}\;{\text{F}}_{i}^{iso}={\text{F}}_{e}^{ani}\;{\text{F}}_{i}^{ani}.$$

The viscous contributions, ${\text{F}}_{i}^{iso}$ and ${\text{F}}_{i}^{ani}$ , define a mapping from the undeformed (reference) configuration to the intermediate configuration of the isotropic part ${\tilde{\Omega }}^{iso}$ for the matrix and the intermediate configuration of the anisotropic part ${\tilde{\Omega }}^{ani}$ for the fibers, respectively (**Figure 3B**). The elastic components ${\text{F}}_{e}^{iso}$ and ${\text{F}}_{e}^{ani}$ are then the complementary mappings from ${\tilde{\Omega }}^{iso}$ and ${\tilde{\Omega }}^{ani}$ , respectively, to the current configuration Ω. It is assumed that the fibers and matrix deform according to the right Cauchy–Green tensor:


(4) $$\text{C}:={\text{F}}^{\text{T}}\text{F}.$$

Analogously we can define the elastic deformation tensors for the isotropic and anisotropic parts


(5) $${\text{C}}_{e}^{iso}:={\text{F}}_{e}^{iso^{T}}{\text{F}}_{e}^{iso}={\text{F}}_{i}^{iso^{-T}}\text{C}{\text{F}}_{i}^{iso^{-1}},$$

(6) $${\text{C}}_{e}^{ani}:={\text{F}}_{e}^{ani^{T}}{\text{F}}_{e}^{ani}={\text{F}}_{i}^{ani^{-T}}\text{C}{\text{F}}_{i}^{ani^{-1}},$$

respectively. To model the transversely isotropic material behavior, we introduce the structure tensor **M**:= **N**⊗**N** in the reference configuration, where **N** is a unit orientation vector pointing in the direction of the fibers. We assume that the orientation vector **N** maps only into the anisotropic intermediate configuration. Thus, we can define the structure tensor (Reese et al., 2021)


(7) $${\tilde{\text{M}}}^{ani}:={\tilde{\text{N}}}^{ani}⊗{\tilde{\text{N}}}^{ani}=\frac{{\text{F}}_{i}^{ani}{\text{MF}}_{i}^{ani^{T}}\;}{{\text{C}}_{i}^{ani}:\text{M}}$$

in the intermediate configuration.

#### 2.4.2 Helmholtz free energy

We assume the existence of a free Helmholtz energy with the property of being an isotropic function of the form $\text{Ψ}\left(\text{C},\text{M},{\text{C}}_{e}^{iso},{\text{C}}_{e}^{ani},{\tilde{\text{M}}}^{ani}\right)$ (see Boehler, 1977; Spencer, 1982; Reese et al., 2001; Reese, 2003). The proposed rheological model (**Figure 3A**) motivates the additive split of this scalar potential into three parts (see Reese and Govindjee, 1998; Nguyen et al., 2007):


(8) $$\text{Ψ}={\text{Ψ}}_{eq}\left(\text{C},\text{M}\right)+{\text{Ψ}}_{neq}^{iso}\left({\text{C}}_{e}^{iso}\right)+{\text{Ψ}}_{neq}^{ani}\left({\text{C}}_{e}^{ani},{\tilde{\text{M}}}^{ani}\right).$$

Here, Ψ_*eq*_(**C**,**M**) represents the equilibrium (rate-independent) response of the matrix and the fibers together, the term ${\text{Ψ}}_{\mathrm{neq}}^{iso}\left({\text{C}}_{e}^{iso}\right)$ the nonequilibrium (time-dependent) response of the matrix, and the term ${\text{Ψ}}_{neq}^{ani}\left({\text{C}}_{e}^{ani},{\tilde{\text{M}}}^{ani}\right)$ the nonequilibrium response of the fibers. We additionally decompose the equilibrium part of the free energy further into isotropic ( ${\text{Ψ}}_{eq}^{iso}\left(\text{C}\right)$ ) and anisotropic parts ( ${\text{Ψ}}_{eq}^{ani}\left(\text{C},\text{M}\right)$ ). This leads to


(9) $$\text{Ψ}={\text{Ψ}}_{eq}^{iso}\left(\text{C}\right)+{\text{Ψ}}_{eq}^{ani}\left(\text{C},\text{M}\right)+{\text{Ψ}}_{neq}^{iso}\left({\text{C}}_{e}^{iso}\right)+{\text{Ψ}}_{neq}^{ani}\left({\text{C}}_{e}^{ani},{\tilde{\text{M}}}^{ani}\right).$$

The stress response of the matrix is assumed to be nearly incompressible. To model such behavior, the following Neo-Hookean free energy density for the matrix is considered:


(10) $${\text{Ψ}}_{eq}^{iso}\left(\text{C}\right)=\frac{\mu_{eq}^{iso}}{2}\left(\text{tr}\;\text{C}-3-\ln(\det\;\text{C})\right)+\frac{\Lambda_{eq}^{iso}}{4}\left(\det\;\text{C}-1-\ln(\det\;\text{C})\right).$$

For the nonequilibrium contribution of the free energy for the matrix the energy


(11) $${\text{Ψ}}_{neq}^{iso}\left({\text{C}}_{e}^{iso}\right)=\frac{\mu_{neq}^{iso}}{2}\left(\text{tr }{\text{C}}_{e}^{iso}-3-\ln(\det\;{\text{C}}_{e}^{iso})\right)+\frac{\Lambda_{neq}^{iso}}{4}\left(\det\;{\text{C}}_{e}^{iso}-1-\ln(\det\;{\text{C}}_{e}^{iso})\right)$$

is assumed. For the equilibrium contribution of the fibers we choose (see Holzapfel et al., 2000)


(12) $${\text{Ψ}}_{eq}^{ani}\left(\text{C},\text{M}\right)=\frac{K_{1,eq}^{ani}}{2K_{2,eq}^{ani}}\left(\exp\;\left[K_{2,eq}^{ani}{\left(\text{tr }(\text{CM})-1\right)}^{2}\right]-1\right),$$

and for the nonequilibrium part we choose analogously


(13) $${\text{Ψ}}_{neq}^{ani}({\text{C}}_{e}^{ani},{\tilde{\text{M}}}^{ani})=\frac{K_{1,neq}^{ani}}{2K_{2,neq}^{ani}}\left(\exp\;\left[K_{2,neq}^{ani}{\left(\text{tr }({\text{C}}_{e}^{ani}{\tilde{\text{M}}}^{ani})-1\right)}^{2}\right]-1\right).$$

The quantities $\mu_{eq}^{iso}$ , ${\text{Λ}}_{eq}^{iso}$ , $\mu_{neq}^{iso}$ , ${\text{Λ}}_{neq}^{iso}$ , $K_{1,eq}^{ani}$ , $K_{2,eq}^{ani}$ , $K_{1,neq}^{ani}$ , and $K_{2,neq}^{ani}$ are material constants which have to be fitted to the experimental results presented in section 3.2.

#### 2.4.3 Dissipation inequality

The constitutive equations are needed to determine the global system of equations. For thermodynamic consistency, the internal dissipation inequality


(14) $$\frac{1}{2}\text{S}:\dot{\text{C}}-\dot{\text{Ψ}}\geq 0$$

has to be fulfilled. Here, **S**=**F** ^−1^ **P** is is the second Piola-Kirchhoff stress tensor. Inserting the Helmholtz free energy (9) into the Clausius-Duhem inequality (14) and considering the fact that **M** is constant, yields


(15) $$\frac{1}{2}\text{S}:\dot{\text{C}}-\frac{\partial \text{Ψ}}{\partial \text{C}}:\dot{\text{C}}-\frac{\partial \text{Ψ}}{\partial {\text{C}}_{e}^{iso}}:{\dot{\text{C}}}_{e}^{iso}-\frac{\partial \text{Ψ}}{\partial {\text{C}}_{e}^{ani}}:{\dot{\text{C}}}_{e}^{ani}-\frac{\partial \text{Ψ}}{\partial {\tilde{\text{M}}}^{ani}}:{\dot{\tilde{\text{M}}}}^{ani}\geq 0\;.$$

Further, we define the rate tensors ${\dot{\text{C}}}_{e}^{iso}$ , ${\dot{\text{C}}}_{e}^{ani}$ and the time derivative of ${\tilde{\text{M}}}^{ani}$ (Reese, 2003; Reese and Christ, 2008) by means of


(16) $${\dot{\text{C}}}_{e}^{iso}=-l_{i}^{iso^{T}}{\text{C}}_{e}^{iso}+{\text{F}}_{i}^{iso^{-T}}\dot{\text{C}}{\text{F}}_{i}^{iso^{-1}}-{\text{C}}_{e}^{iso}l_{i}^{iso},$$

(17) $${\dot{\text{C}}}_{e}^{ani}=-l_{i}^{ani^{T}}{\text{C}}_{e}^{ani}+{\text{F}}_{i}^{ani^{-T}}\dot{\text{C}}{\text{F}}_{i}^{ani^{-1}}-{\text{C}}_{e}^{ani}l_{i}^{ani},$$

(18) $${\dot{\tilde{\text{M}}}}^{ani}=l_{i}^{ani}{\tilde{\text{M}}}^{ani}+{\tilde{\text{M}}}^{ani}l_{i}^{ani^{T}}-2\left({\text{d}}_{i}^{ani}:{\tilde{\text{M}}}^{ani}\right){\tilde{\text{M}}}^{ani}.$$

In the latter equations, the definitions $l_{i}^{iso}:={\dot{\text{F}}}_{i}^{iso}{\text{F}}_{i}^{iso^{-1}}$ , $l_{i}^{ani}:={\dot{\text{F}}}_{i}^{ani}{\text{F}}_{i}^{ani^{-1}}$ have been used. The tensor ${\text{d}}_{i}^{ani}$ is the symmetric part of $l_{i}^{ani}$ . Exploiting the symmetry of $\partial \text{Ψ}/\partial {\text{C}}_{e}^{iso}$ and $\partial \text{Ψ}/\partial {\text{C}}_{e}^{ani}$ , the Clausius-Duhem inequality (15) can be rewritten to read


(19) $$\left(\text{S}-2\frac{\partial \Psi }{\partial \text{C}}-2{\text{F}}_{i}^{iso^{-1}}\frac{\partial \Psi }{\partial {\text{C}}_{e}^{iso}}{\text{F}}_{i}^{iso^{-T}}-2{\text{F}}_{i}^{ani^{-1}}\frac{\partial \Psi }{\partial {\text{C}}_{e}^{ani}}{\text{F}}_{i}^{ani^{-T}}\right):\frac{1}{2}\dot{\text{C}}+{\text{Σ}}^{iso}:l_{i}^{iso}+\Gamma^{ani}:l_{i}^{ani}\geq 0.$$

Here, Σ^*iso*^ and **Γ** ^*ani*^ are symmetric tensors (Svendsen, 2001) defined by


(20) $${\text{Σ}}^{\mathrm{iso}}:=2{\text{C}}_{e}^{iso}\frac{\partial \Psi }{\partial {\text{C}}_{e}^{iso}},$$

(21) $$\Gamma^{ani}:=2{\text{C}}_{e}^{ani}\frac{\partial \Psi }{\partial {\text{C}}_{e}^{ani}}-2\frac{\partial \Psi }{\partial {\tilde{\text{M}}}^{ani}}{\tilde{\text{M}}}^{ani}+2\frac{\partial \Psi }{\partial {\tilde{\text{M}}}^{ani}}:\left({\tilde{\text{M}}}^{ani}⊗{\tilde{\text{M}}}^{ani}\right).$$

#### 2.4.4 Constitutive equations

Following the Coleman-Noll procedure (see Coleman and Noll, 1961) the inequality (19) will be sufficiently satisfied if the second Piola-Kirchhoff stress tensor *S* is given by the relation


(22) $$\text{S}=2\frac{\partial \text{Ψ}}{\partial \text{C}}+2{\text{F}}_{i}^{iso^{-1}}\frac{\partial \text{Ψ}}{\partial {\text{C}}_{e}^{iso}}\;{\text{F}}_{i}^{iso^{-T}}+2{\text{F}}_{i}^{ani^{-1}}\frac{\partial \text{Ψ}}{\partial \;{\text{C}}_{e}^{ani}}\;{\text{F}}_{i}^{ani^{-T}}.$$

Further, the inequality ${\text{Σ}}^{iso}:{\text{d}}_{i}^{iso}+\Gamma^{ani}:{\text{d}}_{i}^{ani}\geq 0$ has to hold. This can be achieved by making the following choices for the evolution equations:


(23) $${\text{d}}_{i}^{iso}=\frac{1}{\tau^{iso}}\frac{\partial g^{iso}\left(\Sigma^{iso}\right)}{\partial \Sigma^{iso}},$$

(24) $${\text{d}}_{i}^{ani}=\frac{1}{\tau^{ani}}\frac{\partial g^{ani}\left(\Gamma^{ani}\right)}{\partial \Gamma^{ani}}.$$

Here, *τ* ^*iso*^ and *τ* ^*ani*^ are relaxation times, and the quantities *g* ^*iso*^ and *g* ^*ani*^ are relaxation potentials. The potential of the isotropic part *g* ^*iso*^ can be additively decomposed into isochoric and the volumetric parts according to


(25) $$g^{iso}({\text{Σ}}^{iso}):=\frac{1}{2\mu_{neq}^{iso}}\text{tr }\left({\left({\text{dev Σ}}^{iso}\right)}^{2}\right)+\frac{1}{9\kappa_{neq}^{iso}}\text{tr }{\left({\text{Σ}}^{iso}\right)}^{2},$$

where $\kappa_{neq}^{iso}:={\text{Λ}}_{neq}^{iso}+2/3\mu_{neq}^{iso}$ represents the bulk modulus. The relaxation potential of the anisotropic part *g* ^*ani*^ describes the stresses in the direction of the fibers and is defined by


(26) $$g^{ani}(\Gamma^{ani}):=\frac{1}{2K_{1,neq}^{ani}}\text{tr }{\left(\Gamma^{ani}\text{sym }({\text{C}}_{e}^{ani}{\tilde{\text{M}}}^{ani})\right)}^{2}.$$

To represent all the constitutive equations in the reference configuration, the evolution equations (23) and (24) need to be pulled back to the reference configuration. A detailed description of this procedure is beyond the scope of this work – the interested reader is referred to e.g. (Marsden and Hughes, 1994; Reese and Christ, 2008). The pull-back leads to the evolution equations


(27) $$\begin{array}{cccc} {\dot{\text{C}}}_{i}^{iso}=2 & {\text{F}}_{i}^{iso^{T}} & {\text{d}}_{i}^{iso} & {\text{F}}_{i}^{iso} \end{array}=\frac{1}{\tau^{iso}}{\text{f}}^{iso},$$

(28) $$\begin{array}{cccc} {\dot{\text{C}}}_{i}^{ani}=2 & {\text{F}}_{i}^{ani^{T}} & {\text{d}}_{i}^{ani} & {\text{F}}_{i}^{ani} \end{array}=\frac{1}{\tau^{ani}}{\text{f}}^{ani},$$

where **f** ^*iso*^ and **f** ^*ani*^ are defined as


(29) $${\text{f}}^{iso}:=2{\text{F}}_{i}^{iso^{T}}\frac{\partial g^{iso}\left(\Sigma^{iso}\right)}{\partial \Sigma^{iso}}{\text{F}}_{i}^{iso},$$

(30) $${\text{f}}^{ani}:=2{\text{F}}_{i}^{ani^{T}}\frac{\partial g^{ani}\left(\Gamma^{ani}\right)}{\partial \Gamma^{ani}}{\text{F}}_{i}^{ani}.$$

#### 2.4.5 Numerical implementation

The presented model has been implemented into the software *Matlab* (The MathWorks, Inc., Massachusetts, USA). Software package *AceGen* has been employed to automatically generate source code for the computation of the tangent operators (see e.g. Korelc, 2002; Korelc, 2009; Korelc and Stupkiewicz, 2014).

In summary, at the Gauss point level, the following equations have to be solved:


(31) $$\text{S}=2\frac{\partial \text{Ψ}}{\partial \text{C}}+2{\text{F}}_{i}^{iso^{-1}}\frac{\partial \text{Ψ}}{\partial {\text{C}}_{e}^{iso}}\;{\text{F}}_{i}^{iso^{-T}}+2{\text{F}}_{i}^{ani^{-1}}\frac{\partial \text{Ψ}}{\partial \;{\text{C}}_{e}^{ani}}\;{\text{F}}_{i}^{ani^{-T}},$$

(32) $${\dot{\text{C}}}_{i}^{iso}=\frac{1}{\tau^{iso}}{\text{f}}^{iso},$$

(33) $${\dot{\text{C}}}_{i}^{ani}=\frac{1}{\tau^{ani}}{\text{f}}^{ani}.$$

For the implicit time integration of the evolution equations (32) and (33), the exponential mapping algorithm has been used (see e.g. Reese and Govindjee, 1998; Vladimirov et al., 2008; Lamm et al., 2022). The final form of the update formulas read:


(34) $${\left({\text{C}}_{i}^{iso^{-1}}\right)}_{n}={\text{U}}_{i}^{iso^{-1}}\;\text{exp}\left(\begin{array}{cccc} \frac{\Delta t}{\tau^{iso}} & {\text{U}}_{i}^{iso^{-1}} & {\text{f}}^{iso} & {\text{U}}_{i}^{iso^{-1}} \end{array}\right){\text{U}}_{i}^{iso^{-1}}$$

(35) $${\left({\text{C}}_{i}^{ani^{-1}}\right)}_{n}={\text{U}}_{i}^{ani^{-1}}\;\text{exp}\left(\begin{array}{cccc} \frac{\Delta t}{\tau^{ani}} & {\text{U}}_{i}^{ani^{-1}} & {\text{f}}^{ani} & {\text{U}}_{i}^{ani^{-1}} \end{array}\right){\text{U}}_{i}^{ani^{-1}}$$

where ${\text{U}}_{i}^{iso}:=\sqrt{{\text{C}}_{i}^{iso}}$ and ${\text{U}}_{i}^{ani}:=\sqrt{{\text{C}}_{i}^{ani}}$ are the inelastic right stretch tensors, and Δ*t*: =*t*_*n*+1_−*t*_*n*_ is the time interval.

## 3 Results

### 3.1 Anatomy and morphology

**Figure 4:**
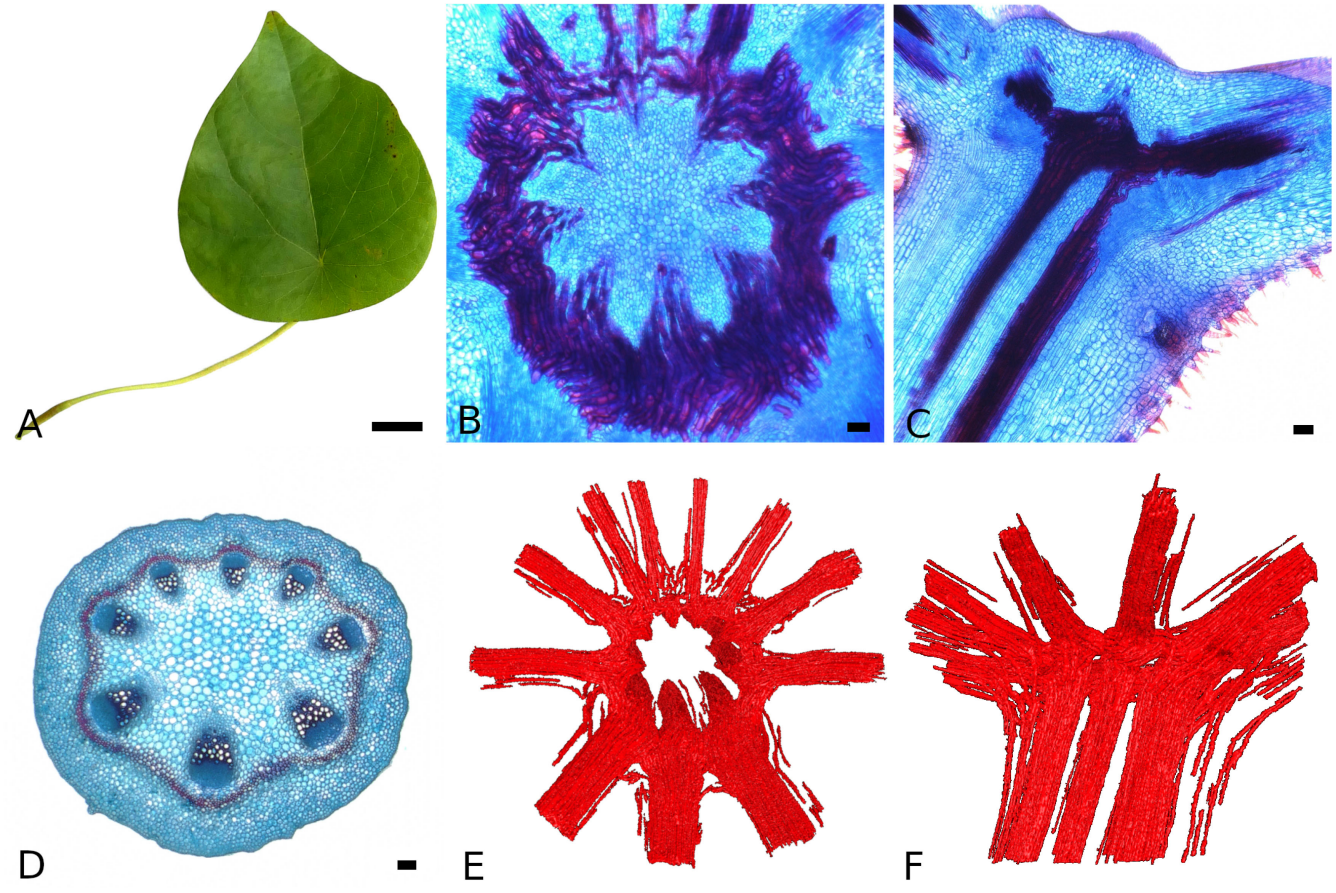
Morphology and anatomy of the *Stephania japonica* leaf. General leaf shape **(A)**, transverse **(B)** and longitudinal section **(C)** of the petiole-lamina transition zone, transverse section of the petiole **(D)** and topview **(E)** and sideview **(F)** of the fiber segmentation from CT data in the transition zone. (for **B**, **D**, **E**: abaxial side at the bottom, staining: astrablue/safranin, scales: 2 cm **(A)** and 100 µm **(B-D)**.

The analyzed leaves of *S. japonica* (**Figure 4A**) show the triangular-ovate shape described for this species and an average lamina length of 14.9±1.6 cm and width of 12.3±1.3 cm. The average lamina area is 120.2±23 cm^2^. The petiole is 2.5±0.2 mm in diameter and 11.8±2.7 cm long. The petiole is inserted eccentric with an average distance of the petiole insertion point from the basal leaf margin of 3.0±0.5 cm. The lamina is palmately veined. From the petiole insertion point, the main vein runs towards the leaf apex. Larger veins run towards the leaf sides, while smaller veins supply the base of the leaf, together building the scaffolding of the lamina and surrounding the intercostal areas.

The petiole shows a more or less circular shape in cross-section (**Figure 4D**). The vascular bundles are arranged in a circle with the xylem positioned towards the center and the phloem towards the periphery. The bundles on the abaxial side are larger than those on the adaxial side. Sclerenchymatous tissue accompanies the vascular bundles on the phloem side. This tissue can be arranged as sclerenchymatous caps or in a closed ring. The percentage of lignified tissue in the petiole averages at 7.2±3.4 *%* . Statistical analysis (Mann-Whitney U test) shows, that the basal part of the petiole has significant higher percentages than the apical part ( 10.6±2.4 *%* vs. 3.5±1.0 *%*).

The petiole-lamina transition zone shows a ring-like structure (**Figures 4B, C**). The xylem bundles run parallel in the petiole and from there into the transition zone where each bundle cross-links and merges with at least the neighboring bundle to form a new bundle that runs into a vein of the lamina. The three larger bundles from the abaxial side of the petiole run into the transition zone and subsequently, form the three largest veins in the lamina. The cross-linking in the transition zone is achieved by single xylem elements from one bundle connecting to xylem elements of the neighboring bundles (**Figures 4B, E**). The additional sclerenchymatous fibers associated with the vascular bundles run into the transition zone and into the veins of the lamina accompanying the vascular bundles (**Figure 4F**).

### 3.2 Mechanical tests

**Figure 5:**
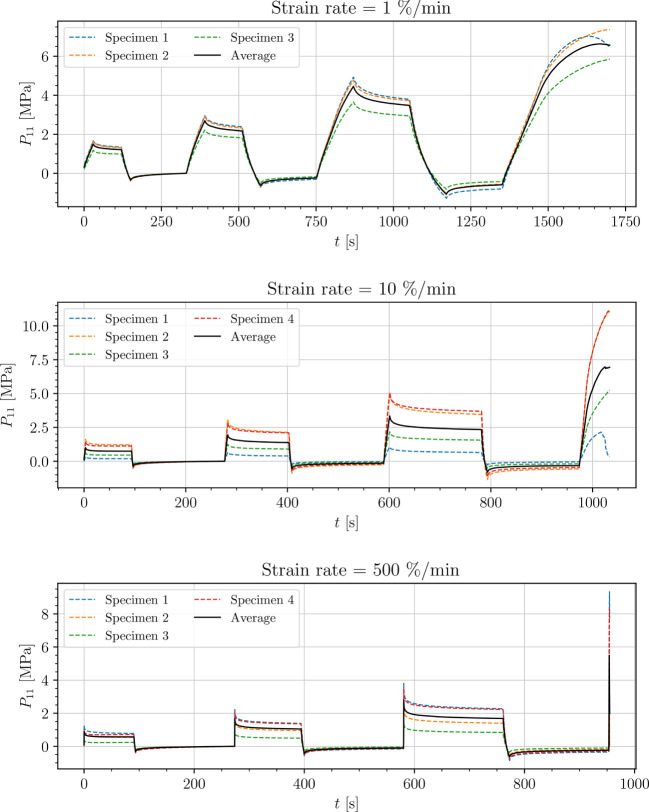
Uniaxial cyclic tensile stress-relaxation test of petiole.

**Figure 6:**
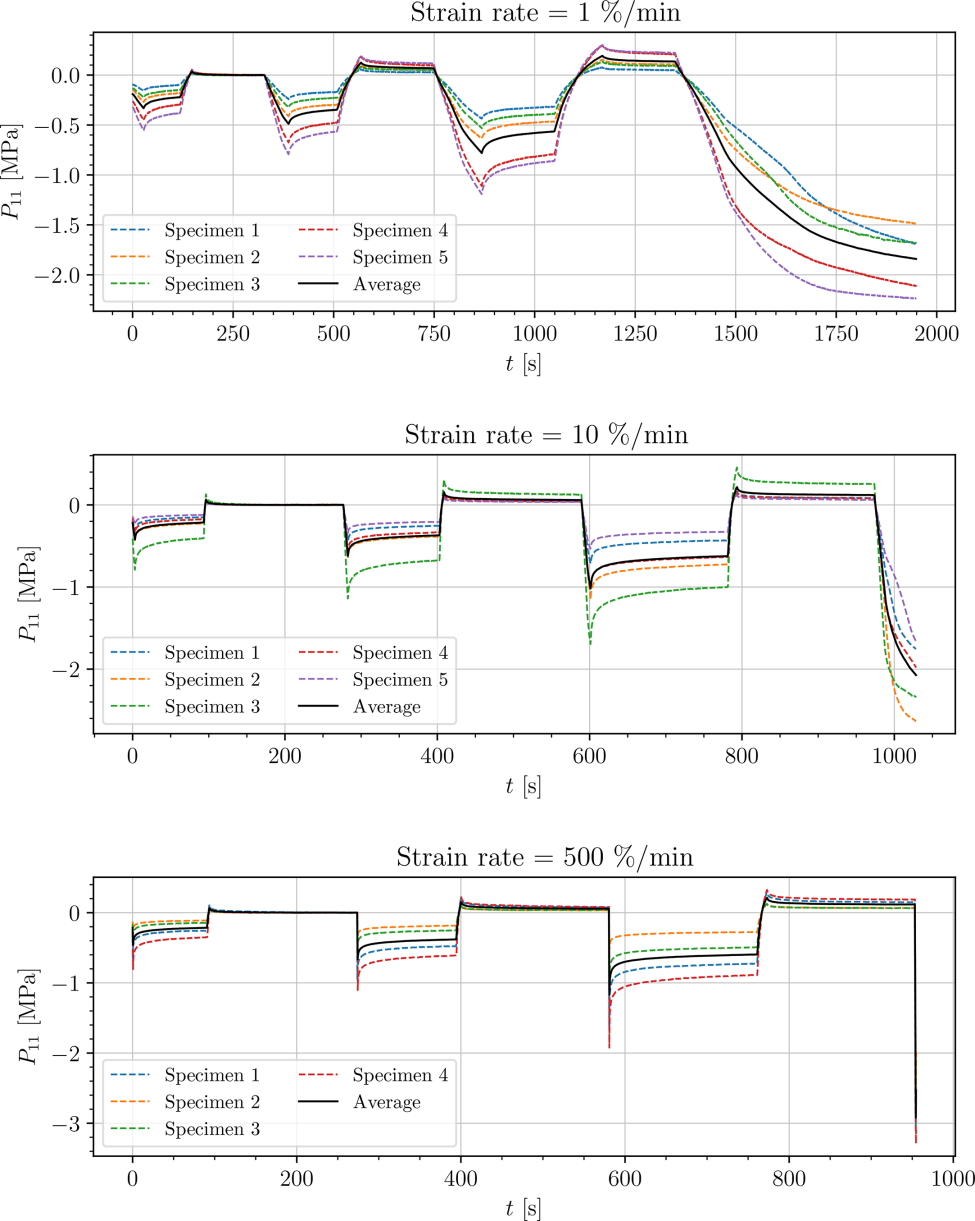
Uniaxial cyclic compression stress-relaxation test of petiole.

**Figure 7:**
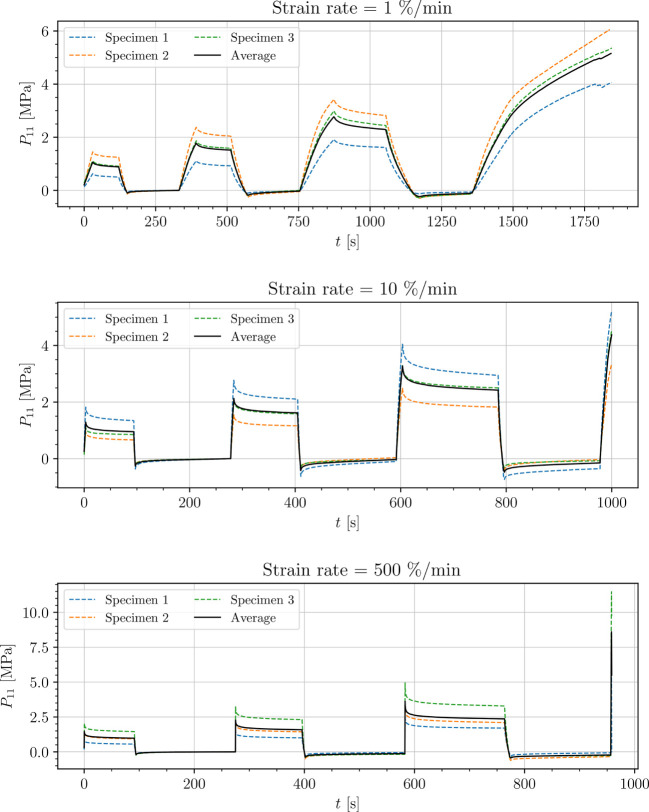
Uniaxial cyclic tensile stress-relaxation test of venation.

**Figure 8:**
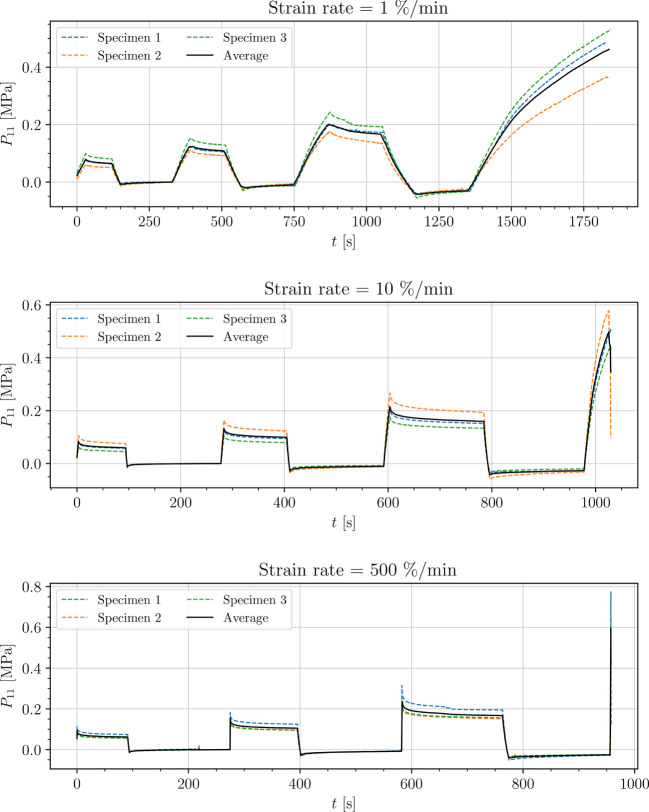
Uniaxial cyclic tensile stress-relaxation test of intercostal areas.

The stress-time curves of cyclic uniaxial tensile experiments are plotted in **Figures 5**, **7**, **8** for the petiole, venation and intercostal area, respectively. Furthermore, stress-time curves from uniaxial compression tests of the petiole are plotted in **Figure 6**.

Results show a clear viscoelastic behavior of the petiole, venation and intercostal area. At higher engineering strain rates tissues respond with increased stiffness (**Figures 5**–**8**). After the loading the stress relaxes to the equilibrium state. In the results we also see, that equilibrium states are independent of the engineering strain rate. The equilibrium states at fixed engineering strains 0.5 %, 1.0 % and 2.0 % are listed in **Table 1**. Petiole and venation show a similarly stiff response. Intercostal area responds to tension with a ten times lower stiffness than the petiole and venation (see **Table 1**). The equilibrium strain-stress response is nonlinear (see **Table 1**).

**Table 1:** Equilibrium state of each fixed strain.

	${\left(P_{11}^{eq}\right)}_{0.5}$ [MPa]	${\left(P_{11}^{eq}\right)}_{1.0}$ [MPa]	${\left(P_{11}^{eq}\right)}_{2.0}$ [MPa]
Petiole: tension	0.8436	1.5344	2.5054
Petiole: compression	-0.2169	-0.3661	-0.5917
Venation: tension	0.9400	1.5738	2.3623
Intercostal area: tension	0.0622	0.1043	0.1645

All tests (**Figures 5**–**8**) show, that in the second and third cycle when the engineering strain has been removed, the material does not recover to the initial state.

### 3.3 Simulation

The simulation of the uniaxial cyclic tensile tests of the petiole, venation, intercostal area, and the uniaxial compression test of the petiole of *S. japonica* were performed at a material point level. The implemented material model includes 10 parameters introduced in Section 2.4, which have to be identified beforehand. This can be achieved in different ways. We decided for the curve fitting approach *via* optimization, where we used an algorithm, which finds the parameters by minimizing the squared difference between the experimental data and the simulation results.

The parameters of the model were fitted only to the averaged stress-time curves of the tests where the loading with strain rate of 10 *%* min^-1^ was applied (**Figure 2**). Furthermore, the fits are restricted to uniaxial tension, and by petiole also to uniaxial compression. All the identified parameters are listed in **Table 2**.

**Table 2:** Fitted parameters of the transversely isotropic viscoelastic material model.

	Petiole: tension	Petiole: compression	Venation: tension	IC: tension
$\mu_{eq}^{iso}$ [MPa]	5.4636	4.5772	3.4486	1.3677
${\text{Λ}}_{eq}^{iso}$ [MPa]	76.9086	84.3337	117.8530	124.7529
$\mu_{neq}^{iso}$ [MPa]	17.3150	5.7484	8.6313	0.8739
${\text{Λ}}_{neq}^{iso}$ [MPa]	9.4537	12.5273	9.9861	19.0484
$K_{1,eq}^{ani}$ [MPa]	21.3795	3.9487	27.9664	1.1358
$K_{2,eq}^{ani}$ [MPa]	0.0163	0.0230	0.0274	0.0306
$K_{1,neq}^{ani}$ [MPa]	21.0270	20.6416	24.3466	3.4247
$K_{2,neq}^{ani}$ [MPa]	6.7858	8.4126	10.8627	20.0358
1/*τ* ^*iso*^ [s^-1^]	0.0046	0.0083	0.0004	0.0121
1/*τ* ^*ani*^ [s^-1^]	0.0641	0.0938	0.0566	0.4246

The Lamé parameters $\mu_{eq}^{iso}$ and ${\text{Λ}}_{eq}^{iso}$ can be expressed as


(36) $$\mu_{eq}^{iso}=\frac{E_{eq}^{iso}}{2\left(1+\nu_{eq}^{iso}\right)},$$

(37) $${\text{Λ}}_{eq}^{iso}=\frac{\nu_{eq}^{iso}E_{eq}^{iso}}{\left(1-2\nu_{eq}^{iso}\right)\left(1+\nu_{eq}^{iso}\right)},$$

where $E_{eq}^{iso}$ is a Young’s module of the isotropic part and $\nu_{eq}^{iso}$ is the corresponding Poisson’s ratio. If we assume, that the Poisson’s ratios of the isotropic part (matrix) of all three structural components of the leaf of *S. japonica* (petiole, venation and intercostal area) are the same, we can compute its average value from the fitted parameters to read ${\bar{v}}_{iso}$ = 0.480. This result can be used in further computations.

The fibers in soft tissues usually cannot carry compressive loadings. Due to this fact, a clear tension-compression asymmetry is observed in the results. The obtained petiole parameters for tension differ from those for compression. If we compute the Young’s modulus of the matrix part $E_{eq}^{iso}$ for the petiole using eq. (36) and (37), we obtain an averaged value $E_{eq}^{iso}=16.029\;\text{MPa}$ for tension and $E_{eq}^{iso}=13.496\;\text{MPa}$ for compression. The $E_{eq}^{iso}$ values for tension for venation and intercostal areas averaged 10.248 MPa and 4.088 MPa, respectively. Hence, petiole possesses the highest and intercostal areas the lowest tensile stiffness of the isotropic part of the tissue (matrix).

The anisotropic constants $K_{1,eq}^{ani}$ determine the equilibrium stiffness of the anisotropic part of the tissues (fibers). Our results show that venation possesses a higher $K_{1,eq}^{ani}$ value than petiole (see **Table 2**). This corresponds to the fact that venation consists mainly of xylem fibers (see Sec. 3.1). The $K_{1,eq}^{ani}$ value of intercostal area is very low, which also corresponds to its anatomy (see Sec. 3.1). Thus, the anisotropic part of the intercostal area can be neglected in future computations. $K_{1,eq}^{ani}$ constants determine the nonequilibrium stiffness of the anisotropic part. The higher they are, the higher the extra (nonequilibrium) stress at some constant strain rate. Accordingly, the petiole and the venation show a similar amount of relaxed stress.

Viscous effects in the material are distributed between the isotropic and the anisotropic parts of the tissue. The parameters 1/*τ* ^*iso*^ and 1/*τ* ^*ani*^ are directly related to the viscosity of the material which influences the nonequilibrium stress. From our results, it is evident that the anisotropic parts (fibers) have a lower relaxation time than the isotropic parts. This means that fibers relax faster than the matrix.

**Figure 9:**
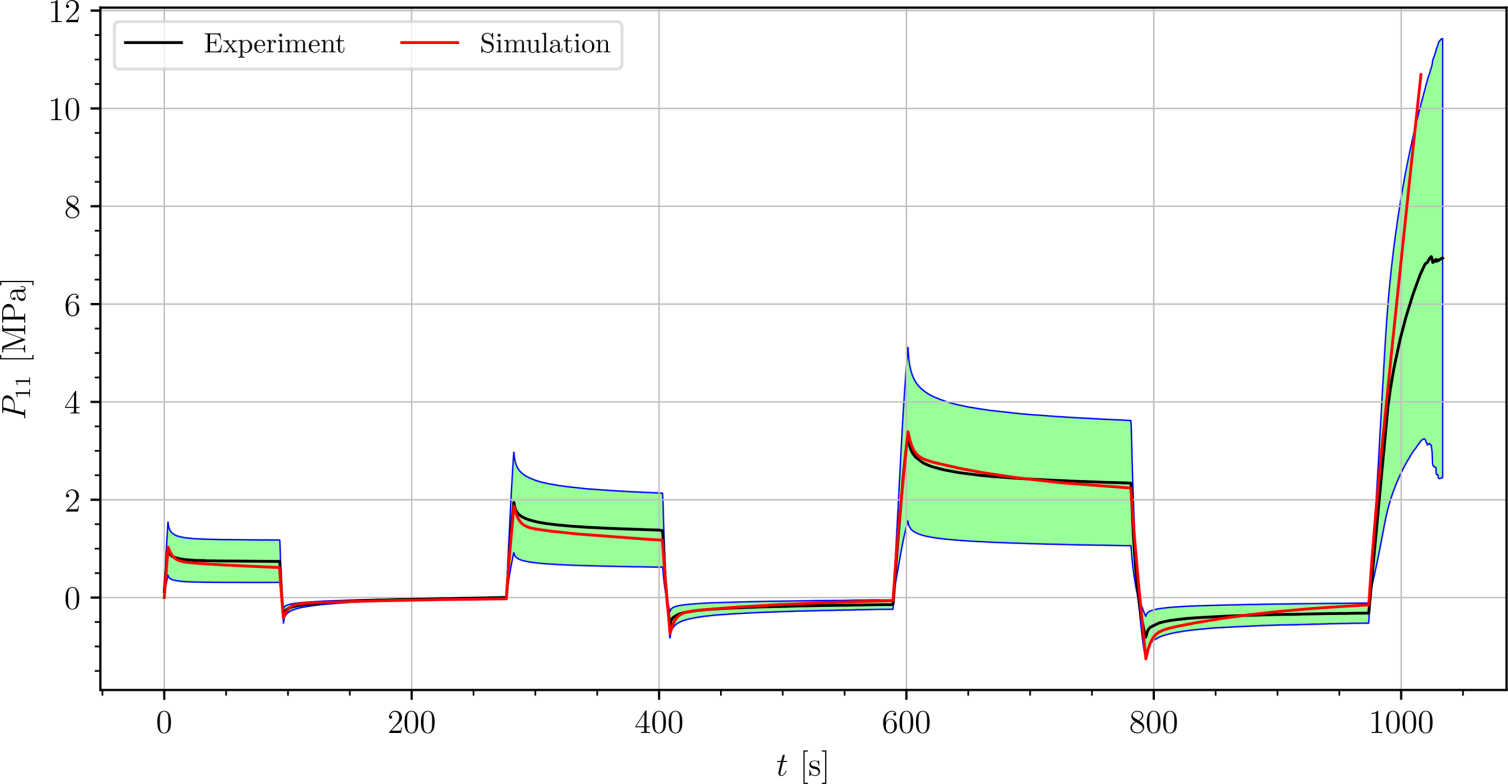
Simulation of the cyclic uniaxial tensile stress-relaxation test of the petiole (red curve). Green region indicates a standard deviation from the averaged experimental result (black curve).

**Figure 10:**
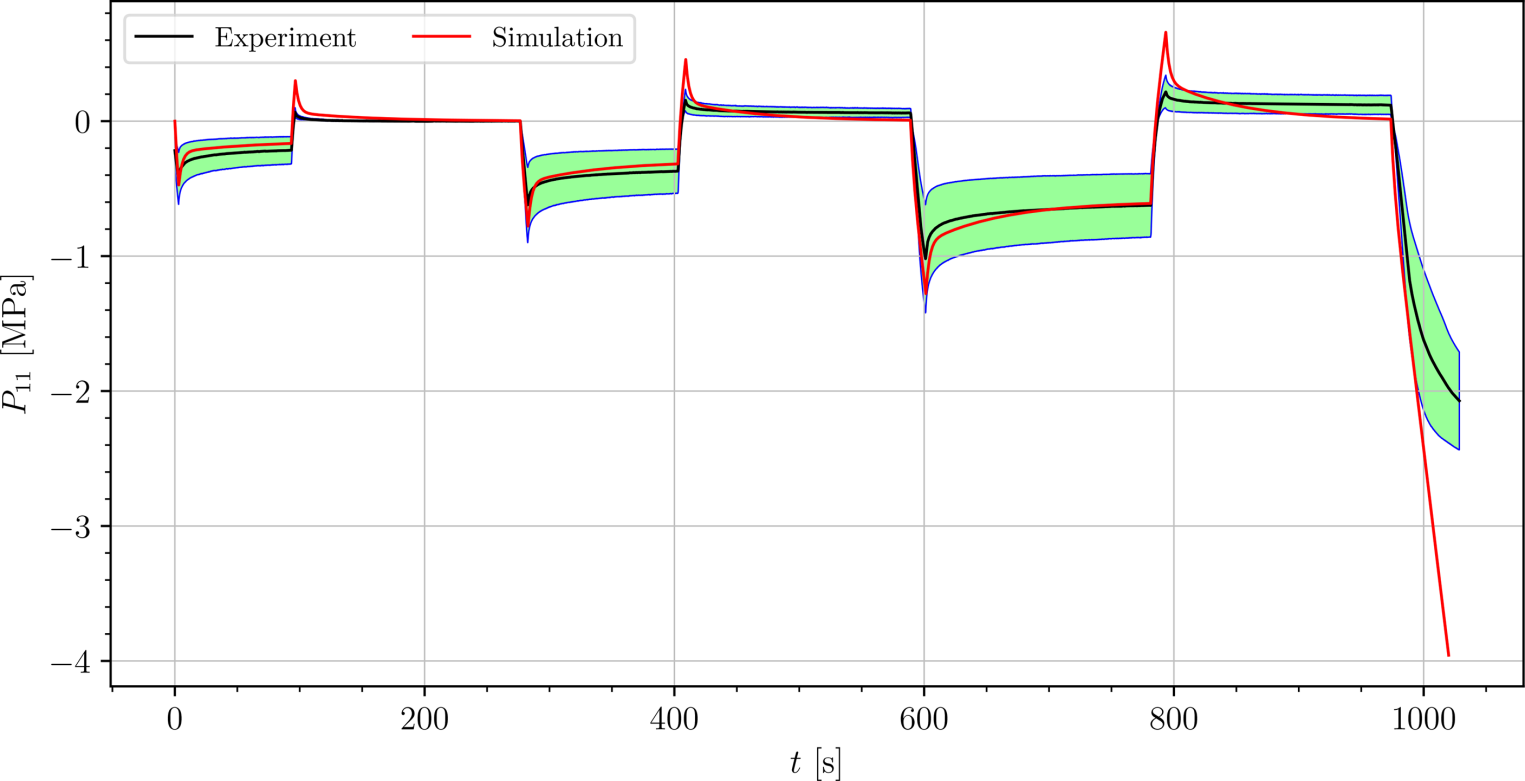
Simulation of the cyclic uniaxial compression stress-relaxation test of the petiole (red curve). Green region indicates a standard deviation from the averaged experimental result (black curve).

**Figure 11:**
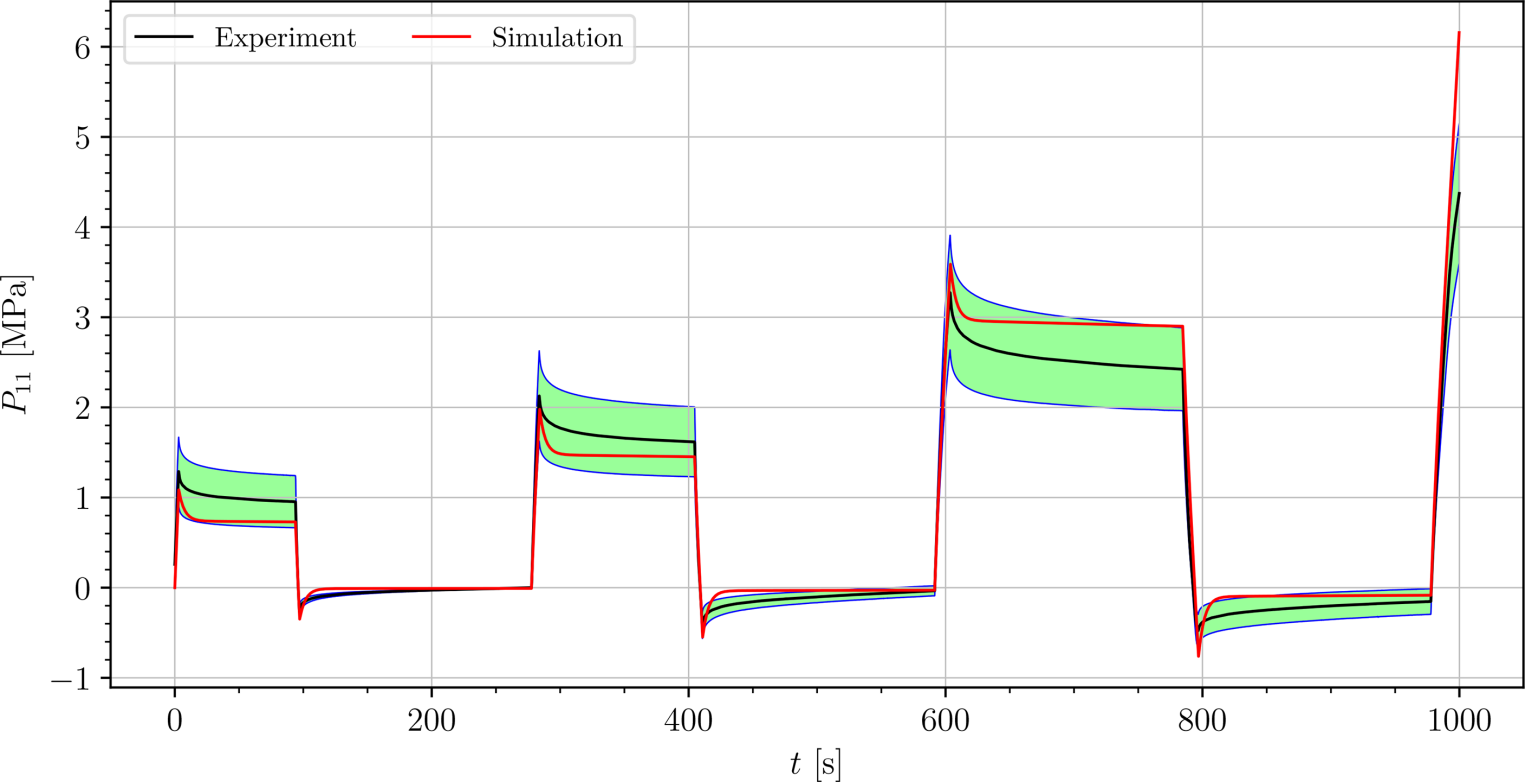
Simulation of the cyclic uniaxial tensile stress-relaxation test of the venation (red curve). Green region indicates a standard deviation from the averaged experimental result (black curve).

**Figure 12:**
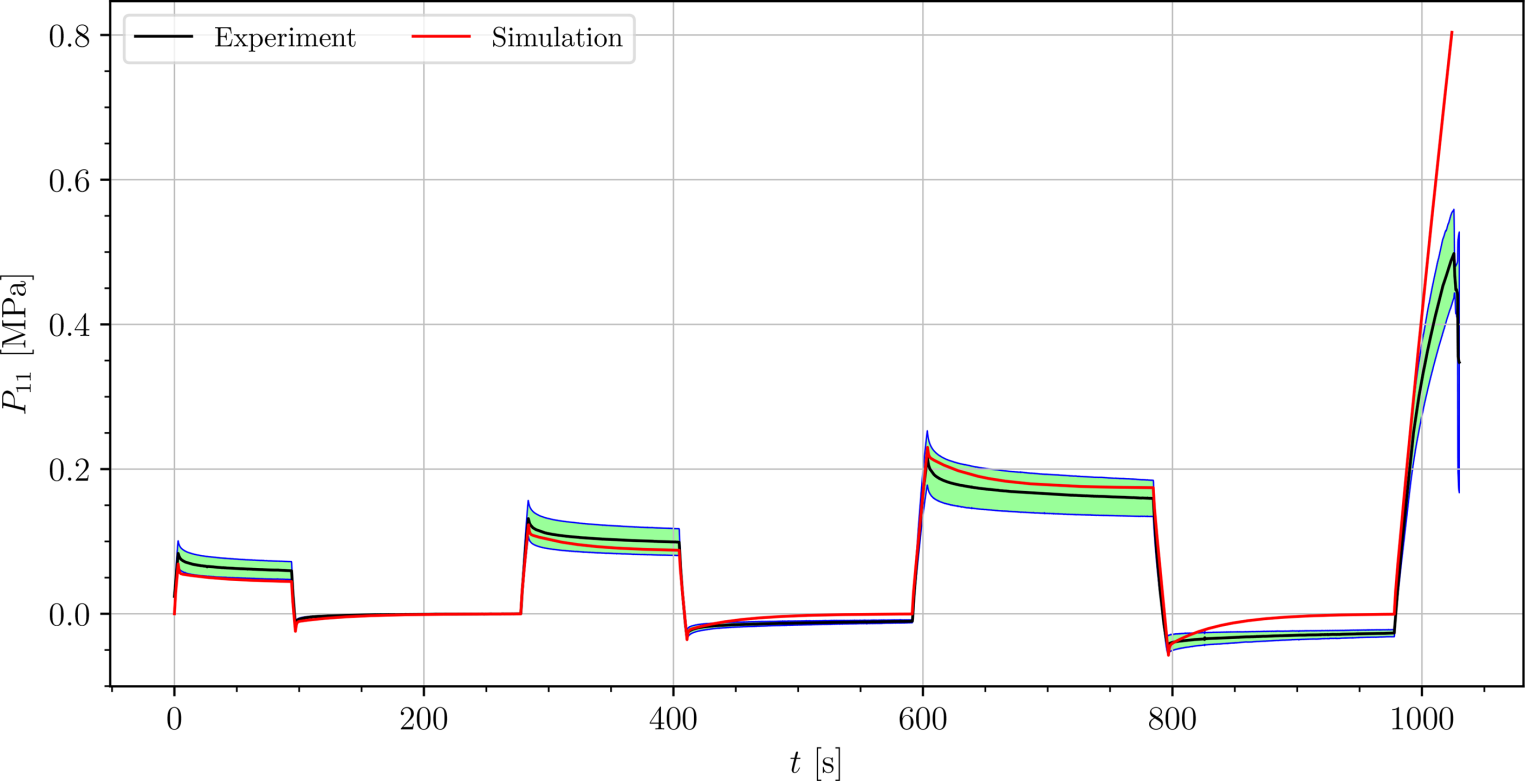
Simulation of the cyclic uniaxial tensile stress-relaxation test of the intecostal area (red curve). Green region indicates a standard deviation from the averaged experimental result (black curve).

We simulated four different cyclic stress-relaxation experiments, where the 10 % min^−1^ strain rate has been applied for loading and unloading. The strain was applied in the same way as it was applied in the experiments - three cycles of loading, holding and unloading (see **Figure 2**). The simulated results are plotted in the **Figures 9**–**12**. Black curves represent averaged experimental results, red curves represent simulation results and the green regions indicate a standard deviation from the averaged experimental results.

The simulated results of the tensile test of petiole stays well within the experimental error. Simulations of tensile tests of venation and intercostal area still show a good match but deviate slightly from the experimental data, especially after the unloading. The reason for this is that after each unloading, viscoelastic material should theoretically relax to a zero line. This different behavior of the samples has been already observed in Section 3.2. The reason for this is explained in Section 4.

To compare the experimental and simulation results, we define a normalized root-mean-square error (*NRMSE* )


(38) $$NRMSE:=\frac{1}{P_{11}^{exp}{\;}_{max}-P_{11}^{exp}{\;}_{min}}\sqrt{\frac{1}{n}\sum_{i=1}^{n}{\left([P_{11}^{sim}{]}_{i}-[P_{11}^{\exp}{]}_{i}\right)}^{2}}$$

where $P_{11}^{exp}{\;}_{max}-P_{11}^{exp}{\;}_{min}$ is the range of the measured data ( $P_{11}^{exp}{\;}_{max}$ is the maximum value, $P_{11}^{exp}\;min$ is the minimum value), *n* is the number of data points, ${\left[P_{11}^{exp}\right]}_{i}$ is the averaged experimental data point for the first component of the first Piola-Kirchhoff stress tensor at *i*-th time, and ${\left[P_{11}^{sim}\right]}_{i}$ is the simulation result at the corresponding time. In **Table 3**, the NRMS errors of the simulated experiments (**Figures 9**–**12**) are listed. In the computation of the error only the result from the beginning of the first to the end of the third cycle were considered. The error stays below 5 % for all four simulations. This result indicates, that the model provides a good fit of the cyclic uniaxial stress-relaxation tests.

**Table 3:** Normalized root-mean-square errors comparing the simulation and experimental data for cyclic uniaxial stress-relaxation tests.

Test	Petiole: tension	Petiole: compression	Venation: tension	IC areas: tension
**NRMSE**	0.0133	0.0323	0.0461	0.0259

## 4 Discussion

The main goal of this study was to investigate the mechanical behavior of peltate leaf of *Stephania japonica*. First, the mechanical tests on petiole, venation and intercostal areas were performed. To arrive at a better understanding of the experimental results a transversely isotropic viscoelastic model for finite deformation was developed and tested. Additionally, the anatomy of petiole and petiole-lamina transition zone was analyzed.

### 4.1 Mechanical tests

The available measurement technique is unfortunately limited in the way that it is not possible to measure Poisson’s ratio. The material model proposed in this work includes ten parameters. The identification of those requires several independent experiments and a challenging testing procedure, which is a research topic in its own. In the future, digital image correlation may help to calibrate the model in a more accurate way, since the whole deformation field can be obtained.

Since only one individuum of *Stephania japonica* was available, the total number of leaves available for testing was limited. This resulted into a relatively small number of samples tested. Furthermore, the samples came from different leaves causing a higher deviation in the experimental results which is not unusual for plant samples. Nevertheless, these results do show the fundamental mechanical behavior of the plant parts. This is sufficient for the calibration of the proposed model. To obtain statistically more accurate results, the sample size should be increased which is left open for future work.

The differences in structure and composition of samples, such as amount of lignin and the degree of lignification, should also be considered in experiments. Since the tests were performed in the first place with the aim to identify the major mechanical mechanisms of plants, only the averaged behavior of plant leaves was taken into account.

### 4.2 Mechanical behavior of the structural elements of the leaf

Mechanical experiments reveal that all parts of the leaf show a rate-dependent behavior. This behavior is typical for plant tissues, especially parenchymatous tissue (Pitt and Chen, 1983; Niklas, 1992) which is, in different proportions, present in all structural elements of the leaf. When parenchyma tissue is stressed, the stresses are redistributed to the cell walls throughout the tissue. The cell walls flatten in reaction to the applied load and fluids from the cell cross the plasma membrane. With higher strain-rates, less time is available for the fluids to diffuse which results in a higher elastic modulus (Niklas, 1992).

Additionally, experimental data (see **Figures 5**–**8**) show that all samples in the second and third cycle, after the strain is removed, do not recover to the initial state (zero stress). This means that all leaf components change their mechanical properties around 1% of strain. This phenomenon can be a result of different processes within the samples. One possibility is occurring damage in the tissues, which is a result of the deformed cell walls due to fluid loss in hydrostatic tissues when loads are applied (as mentioned above), cell–cell debonding or the rupture of cell membrane and walls (Pitt and Chen, 1983; Niklas, 1992; Zdunek and Umeda, 2006). Another reason for this change in mechanical properties could be the change of turgor in the cells as a consequence of water loss during testing since testing times exceeded 15 min and especially in venation and intercostal areas, larger surfaces from cutting were exposed to air. Water loss has a significant impact on the properties of plant tissues including hydrostatic and fibrous tissues. While the stiffness of hydrostatic tissues decreases with water loss, plant fibers usually exhibit a higher stiffness when dried (Niklas, 1992; Niklas, 1999; Faisal et al., 2010). For a clearer understanding of this phenomenon more tests have to be conducted.

Assuming plastic deformation, we can still formulate some statements. Petiole and venation can resist quite high tensile loads – they can withstand around 1 MPa of inner stress at 0.5 % strain (see **Table 1**). As the petiole shows several large xylem fiber bundles, as well as additional sclerenchyma fibers (**Figure 4**) resulting in an average fiber percentage of 7.2 % and the venation consists mostly of vascular tissue, including the lignified xylem fibers, resistance to higher tensile loads is expected. The intercostal areas have a more than ten times lower resistance to tensile stress. This can be attributed to their anatomical features. The intercostal areas of the leaf are mainly composed of spongy parenchymatous mesophyll. Since the stiffness of parenchymatous tissue also depends on the packaging density of the cells and cell-to-cell connections, tissues with a high amount of air-filled spaces like the mesophyll, show only low resistance to mechanical stresses (Gao and Pitt, 1991; Niklas, 1992). The elastic range of the leaf components is below 0.5 % of tensile strain (see **Table 1**). This rather low elastic range, which is typical for solid materials, is also a known phenomenon in hydrostatic plant tissues (Niklas, 1992).

The petiole can withstand around 0.2 MPa pressure at 0.5 % compression strain. In plants, compression strains are mostly carried by tissues that are stronger in compression (e.g., parenchyma) than fibers. Thus, in the petiole of *S. japonica* the tissues bearing these loads are the parenchyma and epidermis.

### 4.3 The petiole-lamina transition zone of the peltate leaf of *Stephania japonica*

The petiole and petiole-lamina transition zone of *S. japonica* showed the same anatomy and fiber arrangement like *S. venosa* and *S. delavayi* (Wunnenberg et al., 2021). Especially the ring-like arrangement of the vascular bundles in the transition zone could be shown in high resolution in this study (**Figure 4**). The segmented CT data allows for understanding of the transition zone on a single-fiber level. Studies have shown the importance of the transition zone in load dissipation and bearing part of the torsional load affecting the leaf (Sacher et al., 2019; Langer et al., 2022). The stability of the transition zone and its ability to dissipate load from lamina and petiole could be achieved by the cross-linking of several xylem elements that branch off of the petiole vascular bundles and merge with elements from the neighboring bundle forming the vascular bundles of the venation. The additional sclerenchyma fibers that run from the periphery of the petiole into the periphery of the leaf veins allow for extra resistance to mechanical loads in the transition zone. Further experiments and modelling could shed further light on the mechanical properties of the petiole-lamina transition zone.

### 4.4 The material model and simulations

We developed a constitutive framework that is capable of predicting an anisotropic viscous behavior (strain-stress curves) of the leaf of *S. japonica*. The fitted parameters are physically meaningful. They give a first insight into plant tissue properties which correspond with experimental results. The simulated results agree well with experimental data. The NRMS errors comparing the experimental and simulation results were less than 5 % for all of the tests.

One of the main goals of this work was to develop and test the proposed continuum mechanical material model. Since the calibration of the model is a highly time consuming process, we identified the material parameters only for the strain rate 10 % min^−1^. This set of parameters is sufficient to test the model. However, it may not be the unique parameter set, which would allow the model to accurately predict any other possible loading rate. To achieve this goal, further calibrations, also in combination with further experiments (e.g. torsion, bending), need to be performed. However, this is beyond the scope of the present contribution.

With this model more complex stress states, experimentally not achievable, can be simulated. Such simulations are very suitable to provide an even better understanding of the behavior of peltate leaves in general and the peltate leaf of *S. japonica*. The model can be easily implemented into a finite element environment. There are some features that can be considered to improve this model, for instance non-constant fiber orientations or temperature dependence of parameters. With a possible extension of this model, not only the response to mechanical loads on petiole, venation and intercostal areas of the leaf can be simulated, but also the complex petiole-lamina transition zone and its mechanical significance in load dissipation. This stays open for future research.

## Data availability statement

The raw data supporting the conclusions of this article will be made available by the authors, without undue reservation.

## Author contributions

DM and AR reviewed the relevant existing literature, planned the experiments and wrote this article. DM performed all simulations and interpreted the experimental and simulation results. AR performed all experiments. HH worked out the theoretical material model and implemented it into the software Matlab (The MathWorks, Inc., Massachusetts, USA). HH, StR, JWS, TL, CN and SR gave conceptual advice, contributed in the discussion of the results, read the article and gave valuable suggestions for improvement. All authors contributed to the article and approved the submitted version.
